# Psychological and behavioral responses to daily weight gain during behavioral weight loss treatment

**DOI:** 10.1007/s10865-024-00476-4

**Published:** 2024-02-26

**Authors:** Charlotte J. Hagerman, Michael C. Onu, Nicole T. Crane, Meghan L. Butryn, Evan. M. Forman

**Affiliations:** 1 Department of Psychological and Brain Sciences, Center for Weight, Eating and Lifestyle Sciences (WELL Center), Drexel University, Stratton Hall, 3201 Chestnut Street, Philadelphia, PA 19104, USA

**Keywords:** Behavioral weight loss, Self-weighing, Weight gain, Motivation, Shame

## Abstract

Self-weighing is consistently associated with more effective weight control. However, patterns show that participants disengage from their weight control behaviors following weight gain. Women with BMIs in the overweight/obese range (N = 50) enrolled in a long-term behavioral weight loss program completed ecological momentary assessment (EMA) surveys immediately after their daily weigh-ins. Nightly EMA surveys and self-monitoring data through Fitbit measured their weight control behavior that day. On days when participants gained weight (vs. lost or maintained), they reported more negative mood, more guilt/shame, and lower confidence in weight control. Motivation following daily weight gain depended on participants’ overall satisfaction with their weight loss so far: more satisfied participants had marginally higher, but less satisfied participants had marginally lower motivation in response to daily weight gain. Greater guilt/shame and lower motivation after the weigh-in predicted less effective weight control behavior that day (e.g., lower likelihood of calorie tracking, fewer minutes of physical activity). Results demonstrate that even small weight gain is distressing and demoralizing for women in BWL programs, which can lead to goal disengagement. These findings have implications for future BWL interventions, including the potential utility of just-in-time adaptive interventions to promote more adaptive responses in the moments after weigh-ins.

## Introduction

Behavioral weight loss (BWL) programs are an integral part of evidence-based treatment strategies for overweight or obesity (Jensen et al., 2014). These interventions facilitate dietary reduction and physical activity promotion by teaching behavioral strategies such as goal setting and problem solving (Wadden et al., 2020), and have strong precedent for producing clinically significant weight loss (~5–10%; Jensen et al., 2014; Wadden et al., 2020). Most BWL programs recommend that participants weigh themselves daily, as regular self-weighing consistently predicts more successful weight control among individuals with BMIs in the overweight/obese range (Brockmann et al., 2020; Shieh et al., 2016; Vuorinen et al., 2021). Although self-weighing has been correlated with higher disordered eating symptomology among adolescents and college-aged populations (Pacanowski et al., 2015), there is strong evidence that this behavior is safe for healthy adults without a history of disordered eating (Benn et al., 2016; LaRose et al., 2014; Pacanowski et al., 2014; Steinberg et al., 2014). Self-weighing during weight loss attempts increases goal salience, heightens awareness of how current eating and exercise behaviors are influencing their weight, and increases personal accountability toward the goal (Patel et al., 2021). During weight loss maintenance, daily self-weighing can bring small weight gains to an individual’s attention immediately, prompting them to take the steps necessary to reverse these small weight gains (e.g., restricting calorie intake, increasing physical activity) before they escalate and become more difficult to address (Butryn et al., 2007).

Nevertheless, studies suggest that participants rarely respond adaptively when they see that they have gained weight. Instead, recent studies have demonstrated clear patterns of goal *disengagement* following weight gain (Frie et al., 2020a; Goldstein et al., 2019; Hayes et al., 2022). For example, Hayes et al. (2022) found that weight regain in a given week predicted less frequent calorie tracking, less frequent self-weighing, less physical activity, and greater weight gain the following week (Hayes et al., 2022). Only very rarely (5% of the time) were participants able to successfully lose weight the week following a gain (Hayes et al., 2022). In other studies that examine temporal patterns of weight change and behavior among people attempting to lose weight, weight gain often precedes a stop in self-weighing altogether (Frie et al., 2020a; Goldstein et al., 2019; Ross et al., 2019a). Taken together, this research suggests that the positive correlation between self-weighing and weight control is at least partially confounded by the fact that participants who gain weight simply stop weighing themselves. To understand how interventions can prevent these patterns of disengagement following weight gain, and to capitalize on the benefits of self-weighing for healthy weight control, it is critical to identify the psychological mechanisms underlying this disengagement. To date, almost no research has attempted this.

### Psychological and behavioral responses to weight gain setbacks

Literature on goal achievement can help explain why participants disengage from their goals following a setback like weight gain. Many lab studies demonstrate that setbacks and perceived failures reduce motivation for a goal by lowering expectations of future success. Participants who are randomly assigned to receive feedback that they failed on a cognitive or other performance task report lower self-efficacy for the task (Smith et al., 2006; Tolli & Schmidt, 2008). They also report lower motivation (Badami et al., 2011; Venables & Fairclough, 2009) and exert less effort in subsequent attempts (Anand et al., 2016; Eskreis-Winkler & Fishbach, 2019). Experiencing setbacks in the form of weight gain is likely to be similarly demoralizing. These setbacks may undermine individuals’ confidence in their ability to control their weight and their motivation for the exceptional effort required to control their weight in the current obesogenic environment (Lakerveld et al., 2018).

Goal disengagement may also occur because weight gain is distressing. Compared to most goals, weight control is particularly fraught, due to pervasive weight stigma that many individuals internalize (Kahan & Puhl, 2017). In qualitative studies, adults with BMIs in the overweight/obese range report that experiencing weight gain is highly upsetting, eliciting frustration as well as the self-conscious emotions of guilt and shame (Frie et al., 2020b; Hartmann-Boyce et al., 2019; Zheng et al., 2018). These negative reactions may be counterproductive to weight control, as negative mood is a common trigger for overeating (Sultson et al., 2022). One study of participants attempting weight loss maintenance found that negative mood in a given week predicted greater likelihood of weight regain that week and the following week (Ross et al., 2019b). Shame in particular has also been shown to increase unhealthy eating (Bottera et al., 2020; Chao et al., 2012). For example, one lab study found that participants randomly assigned to a shame induction (vs. control condition) ate more during a subsequent “taste test” (Chao et al., 2012). Taken together, literature suggests that negative emotional reactions caused by weight gain may hinder an individual’s ability to adaptively respond to the gain.

### Current study

Preliminary evidence suggests that weight gain is distressing and demoralizing to participants, which leads them to disengage from their weight control goals. However, no research to date has systematically tested participants’ immediate psychological responses to weight changes, and whether those psychological responses subsequently influence weight control behavior that day. The current study used ecological momentary assessment (EMA) to test these relationships among women completing a long-term BWL program. This is a secondary data analysis conducted within the context of a larger BWL clinical trial, which allows for a deeper understanding of participant experiences that can help us improve treatment outcomes.

*Aim 1* was to examine how daily weight changes influence immediate cognitions. We hypothesized that (Hypothesis 1a) participants would experience more negative mood, more guilt and shame, lower motivation for weight loss, and lower confidence in controlling their weight on days when they gained weight (versus lost or maintained their weight), and that (Hypothesis 1b) the association of weight gain on negative post-weighing cognitions would be especially strong for participants with lower satisfaction with their weight loss progress thus far. We also examined participants’ open-ended responses to their weighing activity.

*Aim 2* was to test how responses to self-weighing influence self-reported and objective behavior that day. We hypothesized that (Hypothesis 2) more negative mood, more guilt and shame, lower confidence in controlling weight, and lower motivation for weight loss and would lead to worse weight control behavior that day, through self-report (perceptions of that day’s weight control behavior, perceptions of calorie intake) and objective measures (likelihood of calorie tracking and total minutes of moderate-to-vigorous physical activity (MVPA)).

## Methods

### Participants

Participants were 50 women with BMIs in the overweight/obese range enrolled in Cohort 1 of a parent clinical trial, in which they received two years of group-based BWL treatment (for protocol of the parent trial, see Miller et al., 2023). Eligible participants in the parent trial were between 18 and 70 years old, had a BMI ≥ 27, and had no medical or psychiatric conditions that would pose a risk for their participation. The sample in the current study was 72% Non-Hispanic White, 20% Non-Hispanic Black, and 8% White Hispanic (with no other race/ethnicities self-reported), with a mean age of 55.3 (SD = 9.72).

### Procedure

Participants were completing a parent trial that tested the effectiveness of sharing self-monitoring data (i.e., weight, calorie tracking, and physical activity) with various sources of social support (i.e., a coach, a friend/family member, and/or program peers) during a two-year, gold standard BWL program. All participants completed three months of weekly group sessions (through videoconferencing software), followed by monthly coach contact (individual phone calls or group sessions) and monthly coaching messages for the rest of the program. Coaches recommended that participants achieve a 10% weight loss goal, but supported any goal that was healthy, realistic, and sustainable. Participants and coaches collaboratively determined a specific daily calorie goal (between 1200 and 1800 calories) based on the participant’s current weight and activity level, which was periodically adjusted to ensure that participants were losing the targeted 1–2 pounds per week and not struggling with hunger. All participants had the same physical activity goal, which slowly increased (over 12 weeks) to 250 min per week.

All participants were instructed to weigh themselves, track their calories using the Fitbit app, and wear a Fitbit band to track their physical activity on every day of the 24-month program. The current EMA study took place during month nine of the trial, when weight loss tends to slow or be regained (Ostendorf et al., 2021), allowing sufficient variability in weight loss satisfaction and daily weight changes. All participants actively enrolled in Cohort 1 of the parent trial were invited to participate in the study through an email from the study staff. Coaches also described the opportunity during a group session. Those who chose to participate first completed a baseline survey in which they reported their current *satisfaction with their weight loss* on a scale from 1 (not at all satisfied) to 10 (extremely satisfied). For five days in a row, participants received a *post-weighing survey* at 8 am, which they were instructed to complete after they weighed themselves, to assess their current feelings about weight loss. Participants’ *self-monitoring data* in the Fitbit app was used to examine their objective weight control behavior that day. At 9 pm, participants received a *nightly survey* in which they reported their perceptions of their behavior that day. On the final nightly survey (day 5), participants were asked additional questions about their typical responses to self-weighing throughout the program. Participants received $50 for completing all ten surveys, but lost $3 for any survey they did not complete in order to incentivize responses.

### Ecological momentary assessments

Time-contingent ecological momentary assessments were sent two times per day, including a post-weighing survey at 8 am and a nightly survey at 9 pm. See Supplementary Material for a checklist for reporting EMA research (Dao et al., 2021).

#### Post-weighing survey

On the post-weighing survey (completed in the morning), participants first reported their *mood* on a scale from 1 (very bad) to 10 (very good), which was reverse coded so that higher scores indicated more negative mood. *Guilt/shame* was assessed through items with the stem “Right now, my recent eating and exercise behaviors are making me feel…” and “Right now, my weight is making me feel….” Response options were 1 (*very guilty*) to 10 (*very proud*), and 1 (*very ashamed*) to 10 (*very proud*), respectively. Due to their high correlation (r = .81), items were averaged into a single measure of guilt/shame and reversed coded so that higher scores indicated higher guilt/shame.

Participants reported their *confidence in weight control* through their agreement with the statement “I’m feeling confident that if I adhere to my weight loss behaviors, I will lose weight” on a scale from 1 (*strongly disagree*) to 5 (*strongly agree*). If the participant’s goal was to maintain their weight, they were instructed to indicate their confidence in their ability to maintain. Finally, they reported their motivation for weight control with the statement “Right, now it is hard to feel motivated to do some of the things necessary to control my weight, like limit my calories, record my intake, or exercise” on a scale from 1 (*strongly agree*) to 5 (*strongly agree*), which was reverse coded. Finally, participants responded to an *open-ended question* asking them to report how they were feeling about their weight right now.

#### Nightly survey

Relying on participants’ food records as a measure of daily calorie intake is problematic due to large variations in their accuracy and completeness (Carpenter et al., 2022; Schoeller et al., 2013). Instead, to assess participants’ *calorie intake* that day, the nightly EMA survey asked participants to estimate that day’s intake on a scale from 1 (*much higher than my calorie goal*) to 5 (*much lower than my calorie goal*). Scores were reverse coded so that higher scores indicated higher calorie intake. *Self-reported weight control behavior* was measured by asking participants to complete the stem “My eating and exercise behavior today…” with one of the following options: *contributed to weight loss* (1), *kept me at the same weight* (2), or *contributed to weight gain* (3), reverse coded so that higher scores indicated better weight control behavior.

On the final nightly survey (day 5), participants responded to three items that asked them to directly report whether seeing weight gain on the scale makes them feel guilty, makes them feel demoralized, and puts them in a bad mood on a scale from 1 (*strongly disagree*) to 5 (*strongly agree*).

### Self-monitoring data

As part of the BWL program, participants tracked their calories using the Fitbit app and wore a Fitbit band to track their physical activity daily. We measured participants’ *calorie tracking* by recording whether participants had logged at least one food item that day (yes/no). *MVPA* was the total minutes participants spent in the “fairly active” and “very active” minutes that day, i.e., time spent in activity at a metabolic equivalent (MET) of 3 or higher, in bouts of 10 min or more (Semanik et al., 2020).

### Statistical analysis

Analyses were conducted using SAS 9.4 software. Descriptive statistics are provided for all study variables, and within-and between-person correlations were reported. Intraclass correlation coefficients (ICC) are provided for all level-1 variables. Three values of MVPA were excluded from analyses because they were more than 3 standard deviations above the mean. Results that differ when these cases were included are reported in footnotes.

EMA resulted in a nested structure with repeated daily measurements (level-1) for individual participants (level-2). Multilevel analyses were used for all hypothesis tests (Snijders & Bosker, 2011). For all models, missing data was handled using maximum likelihood estimation with the assumption that data are missing at random. Models with continuous outcomes used PROC MIXED and models with the dichotomous outcome (i.e., calorie tracking) were tested using PROC GLIMMIX.^1^ All models controlled for the three experimental conditions of the parent study.

To test Hypothesis 1a, multilevel models examined whether daily weight gain (level 1) predicted post-weighing cognitions (level 1) while accounting for person-level variance. Chi-Square Likelihood Ratio tests determined whether model fit was improved by the inclusion of a random slope. If so, the random slope term was included in the model. We used the same method for models testing Hypothesis 2, in which post-weighing cognitions (level-1) were regressed on daily behaviors (level-1).

To test Hypothesis 1b, we used a cross-level interaction, adding an interaction term to test whether overall weight loss satisfaction (level 2) moderated the association of daily weight gain (level-1) on the post-weighing cognitions. The random slope of the level-1 predictor variable was included (Heisig & Schaeffer, 2019). Weight loss satisfaction was grand mean centered. For significant cross-level interactions, simple slopes were calculated and graphed to depict the slope of the within-person predictor on the outcome of interest at one standard deviation below, above, and at the grand mean of weight loss satisfaction.

To examine open-ended responses to self-weighing (Aim 1), a rater (MO) created a list of themes based on a preliminary review of approximately a quarter of responses, then coded the rest of responses to these themes, adding themes as appropriate. Frequencies for each theme that was mentioned at least 5 times are reported, and exemplar quotations were used to illustrate key findings.

## Results

### Participant characteristics

Seventy-five participants were actively enrolled in Cohort 1 of the parent trial and invited to participate in the current study (only one participant had dropped out of the study at this point). Of these, 59 participants agreed to participate (78.6%). Those who agreed versus declined to participate did not differ by age (*t*=−1.37, *df* = 68, *p* =.176), race (χ^2^ (4, *N* = 75) = 6.29, *p* = .178), baseline BMI (*t*=−1.15, *df* = 73, *p* = .253), or 6-month weight loss (*t*=−1.36, *df* = 68, *p* = .176).

Although the program instructed participants to weigh daily, participants in this and other programs often skip weigh ins (Crane et al., 2023). To achieve an accurate picture of BWL participants’ typical responses to self-weighing, participants were instructed to continue their usual weighing behavior during the study period, resulting in some missed weigh-ins. Five participants did not weigh themselves during the study period and were excluded from analyses. An additional 2 participants could not complete any surveys due to scheduling conflicts, 1 participant’s data could not be matched to their clinical trial data, and 1 male participant was removed so that the sample would be entirely women. The final sample included 50 participants.

At the time of the study, the 50 participants had completed approximately 8.5 months of the program, and had lost an average of 10.1% (*SD* = 7.32) of their body weight. Average self-reported satisfaction with weight loss so far was 6.42 (*SD* = 2.28).

### Daily data

The final 50 participants weighed themselves on an average of 4.50 (SD=1.01) out of 5 days and completed an average of 95.4% of surveys. However, survey responses were excluded if they were completed after 1 pm (for the post-weighing survey) or after 2 am (for the nightly survey), which happened on 16.1% of days when participants weighed in.

See Table 1 for between and within-person correlations and descriptive statistics. Participants gained weight (i.e., ≥ 0.1 pounds) on 45.2% of all weigh-ins on days included in analyses.

#### Hypothesis a: On days that participants gain weight, they will report worse mood, greater guilt/shame, lower confidence in weight control, and lower motivation.

The random slope of daily weight gain was included only for the analysis examining guilt/shame. Consistent with hypotheses, on days when participants gained weight (vs. maintained or lost weight), they subsequently reported more negative mood (*b* = 0.87, *SE* = 0.19, *t* = 4.70, *p* < .001), greater guilt/shame (*b* = 0.85, *SE* = 0.19, *t* = 4.60, *p* < .001), and lower confidence in weight control (*b*=−0.18, *SE* = 0.07, *t*=−2.70, *p* < .01). However, weight gain was not associated with lower motivation to control their weight (*b*=−0.02, *SE*=0.12, *t*=−0.16, *p* = .87). See Supplementary Material for tables reporting full multilevel models testing Hypothesis 1a.

#### Hypothesis b: The association between weight gain and negative post-weighing cognitions will be stronger among participants with lower satisfaction with their weight loss so far.

**Fig. 1:**
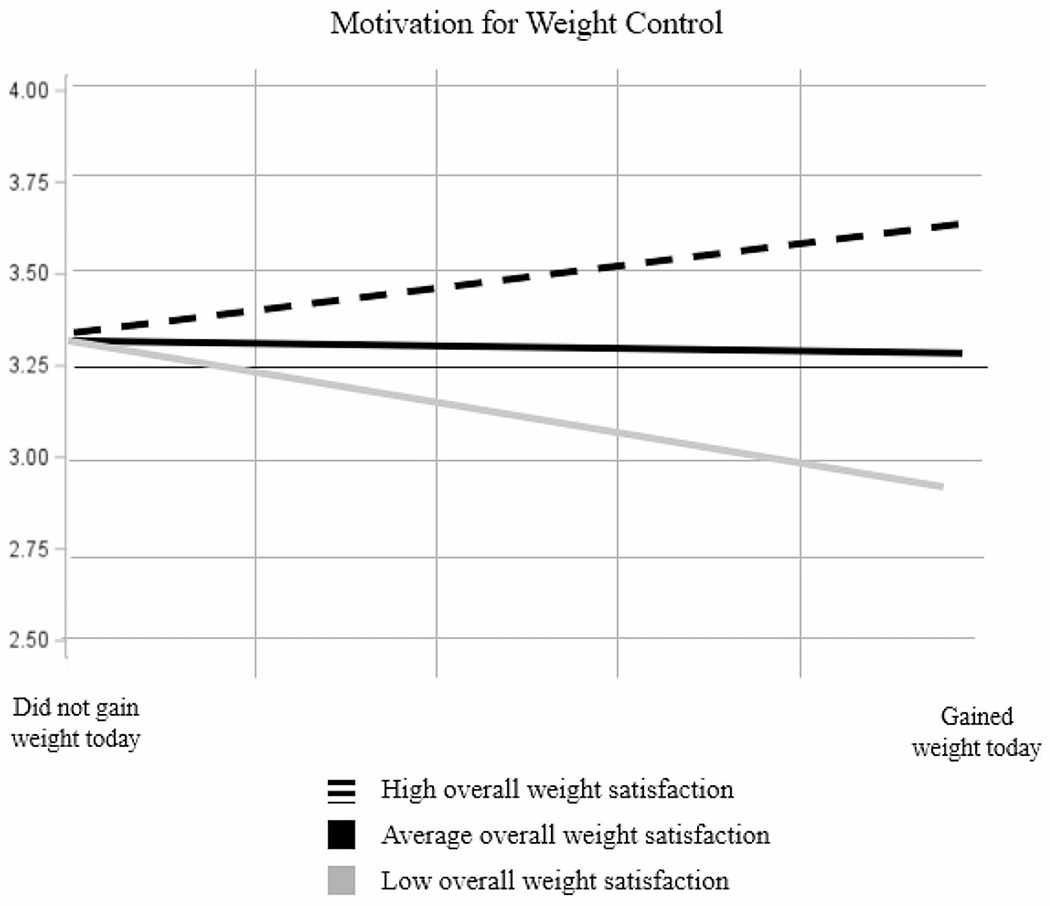
Motivation for weight control in response to daily weight gain among those with low, average, and high satisfaction with their weight control so far

Overall weight satisfaction did not moderate the relationship between weight gain and mood, guilt/shame, or confidence in weight control. However, there was a significant crosslevel interaction between daily weight gain and overall weight loss satisfaction on motivation (*b* = 0.16, *SE* = 0.05, *t* = 2.90, *p* < .01). Simple slopes analysis demonstrated that among participants with lower overall weight satisfaction, daily weight gain led to lower motivation (*b*=−0.41, *SE* = 0.20, *t*=−2.05, *p* = .046). Among participants with average overall weight satisfaction, daily weight gain was not associated with motivation (*b*=−0.05, *SE* = 0.15, *t*=−0.33, *p*=.74). Among those with high overall weight satisfaction, daily weight gain was associated with marginally *more* motivation for weight control (*b* = 0.31, *SE* = 0.19, *t* = 1.67, *p* = .10). See Fig. 1 for a depiction of this interaction. See Supplementary Material for tables of multilevel models testing Hypothesis 1b.

### Open-ended responses to weighing

Frequencies of each theme in response to the question “How do you feel about your weight right now?” and exemplar quotes from each theme are shown in Table 2. Of note, participants spontaneously reported having negative mood more frequently on days when they gained weight, and more frequently reported positive mood on days that they did not gain weight. Responses indicated a neutral mood on less than a quarter of all days. Often, participants reported that their weight trajectory could be better, seeing room for improvement.

On the final day of surveys,

#### Hypothesis 2 Negative post-weighing cognitions will predict lower adherence to weight control behaviors that day.

See Supplementary Material for tables of multilevel models testing Hypothesis 2. The random intercept was retained for all models.

##### Mood.

More negative mood after weighing was not associated with perceptions of weight control behavior that day (*b*=−0.05, *SE* = 0.03, *t*=−1.52, *p* = .13), likelihood of calorie tracking (*b*=−0.08, *SE* = 0.16, *t*=−0.51, *p* = .61), perceived calorie intake (*b* = 0.01, *SE* = 0.05, *t* = 0.18, *p* = .86), but was marginally associated with marginally fewer total minutes of MVPA (*b*=−3.11, *SE* = 1.64, *t*=−1.89, *p* = .06) that day.^2^

##### Guilt/Shame.

On days when participants reported greater guilt/shame after weighing in, they were less likely to track their calories (*b*=−0.44, *SE* = 0.17, *t*=−2.56, *p* = .02); a one-unit increase in guilt/shame was associated with a 35.6% decrease in the likelihood of tracking that day. Guilt/shame was also associated with worse self-reported weight control behavior that day (*b*=−0.09, *SE* = 0.03, *t*=−3.05, *p* < .01) and with lower minutes of MVPA that day (*b*=−5.64, *SE* = 1.75, *t*=−3.22, *p* < .01). However, guilt/shame after weighing was not associated with participants’ perceived calorie intake (*b* = 0.08, *SE* = 0.05, *t* = 1.52, *p* = .13).^3^

##### Confidence in Weight Control.

Confidence in weight control was not associated with a higher likelihood calorie tracking (*b* = 0.62, *SE* = 0.40 *t* = 1.56, *p* = .12), perceptions of behavior that day (*b* = 0.06, *SE* = 0.08, *t* = 0.72, *p* = 47), perceived calorie intake (*b* = 0.03, *SE* = 0.13, *t* = 0.26, *p* = .80), or minutes of MVPA (*b*=−0.41, *SE* = 4.04, *t*=−0.10, *p* = .92) that day.

##### Motivation.

On days when participants reported more motivation for weight control after their daily weigh-in, they were more likely to track their calories that day (*b* = 0.80, *SE* = 0.26, *t* = 3.12, *p* < .01). Each one-unit increase in motivation was associated with an 123% increase in the likelihood of tracking food that day. They also self-reported better weight control behavior (*b* = 0.11, *SE* = 0.05, *t* = 2.27, *p* = .03). However, there was no association between post-weighing motivation and perceived calorie intake (*b*=−0.09, *SE* = 0.08, *t*=−1.14, *p* = .26, and no association with minutes of MVPA that day (*b* = 2.03, *SE* = 2.64, *t* = 0.77, *p* = .44).

## Discussion

Past studies consistently show a pattern in which individuals disengage from their weight control goals after gaining weight (Frie et al., 2020a; Goldstein et al., 2019; Hayes et al., 2022). The current study is among the first to systematically test potential explanations for this pattern. Using EMA, we explored psychological responses to daily weight gain among 50 women with BMIs in the overweight/obese range in month nine of a long-term BWL program, then examined how these responses influence same-day weight control behaviors.

### Responses to weight gain

Participants experienced more negative mood and heightened guilt/shame following a weight gain, relative to maintaining or losing weight. Open-ended responses echo these findings; participants reported negative mood on over a third of the days they gained weight (vs. only about 7% of the days they did not gain weight). Further, when directly asked on the final survey, the majority of participants agreed that weight gain is demoralizing and makes them feel guilty. Together, these results verify previous interviews and focus groups for people with BMIs in the overweight/obese range, who describe weight gain on the scale as a distressing and shameful experience (Frie et al., 2020b; Hartmann-Boyce et al., 2019; Zheng et al., 2016). Our results highlight the societal “moralization” of weight control, which many individuals internalize, experiencing shame about their body and guilt about their health behaviors (Täuber et al., 2018). The meaningful changes in guilt and shame in response to small daily fluctuations in weight demonstrate how self-perceptions are highly contingent on current, ever-changing body satisfaction.

Participants also had decreased confidence in weight control on days they gained weight. This is consistent with broader literature on goal pursuit, which shows that setbacks and perceived failures are demoralizing (Badami et al., 2011; Venables & Fairclough, 2009). However, participants’ motivation following weight gain depended on their overall weight loss satisfaction. Whereas those with low overall satisfaction were demoralized, those with high satisfaction were marginally *more* motivated for weight control after a weight gain. Thus, individuals who are happy with their weight loss may have more motivation to respond to weight gain adaptively, whereas those who are frustrated by their progress may struggle to overcome these setbacks.

### Post-weighing cognitions influence daily behavior

The negative psychological responses to weight gain seen in this study are meaningful and clinically relevant, as they predicted worse adherence to weight control behaviors that same day. Those who experienced more guilt/shame and lower motivation (but not negative mood or confidence in weight control) following a weight gain were less likely to track their calories that day and self-reported worse weight control behavior that day. Greater guilt/shame was also associated with fewer MVPA minutes that day.

Findings are consistent with past evidence that guilt and shame can be maladaptive for health behavior, leading to less healthy eating behavior and avoidance of weight control altogether (Bottera et al., 2020; Chao et al., 2012; Hagerman et al., 2019). Although past research has suggested that guilt and shame play unique roles in behavior—guilt being adaptive, shame being maladaptive (Tangney et al., 2007)—the high correlation between them in the current study suggests that participants may be unable to distinguish their own feelings of guilt and shame on self-report surveys. The fact that behavior was more consistently predicted by guilt/shame than negative mood underscores that self-conscious emotions are uniquely problematic in the context of eating, perhaps because these feelings inspire avoidance.

The finding that low motivation predicts less healthy behavior is intuitive and consistent with past findings. For example, Hayes and colleagues (2022) found that lower motivation (as measured by the perceived importance of “staying on track” compared to other life demands) predicted more weight gain the following week (Hayes et al., 2022). However, it is surprising that confidence in weight control was not associated with any behavioral measure. In retrospect, it would have been apt to include a measure of self-efficacy, i.e., participants’ confidence in their ability to *enact* the weight control behaviors, as this may be more impactful than their confidence in these behaviors’ effectiveness.

It is also unexpected that motivation for weight control was not associated with MVPA minutes. It could be that individuals have a more diverse set of reasons for engaging in physical activity outside of their weight (e.g., mood regulation), making these behaviors less susceptible to disruptions associated with weight-related demoralization. Further, physical activity may be most strongly predicted by existing habits, which persist despite changes in motivation (Wood & Rünger, 2016). Interestingly, none of the post-weighing cognitions were associated with perceived calorie intake that day, perhaps highlighting how difficult it is to accurately estimate personal calorie intake (Schoeller et al., 2013).

### Implications for interventions

This study suggests that participants may need tailored support to guide their response to small weight regain, a common and expected part of long-term weight control. Our results indicate that such support should come immediately, as negative responses to weight gain predict same-day behaviors. Just-in-time adaptive interventions (JITAIs) may be particularly helpful, as they can identify weight gain and provide immediate support (Nahum-Shani et al., 2018). Given that participants struggled with negative affect and shame in particular, participants may need specialized psychological strategies (e.g., mindful and acceptance-based strategies (Forman & Butryn, 2016) to help them cope with the psychological distress of weight gain, rather than solely relying on behavioral techniques to instruct participants how to respond to the weight regain.

### Limitations and future directions

First, analyses were not preregistered because data collection and analyses were expedited to provide pilot data for a specific grant submission with an impending deadline. After having conducted preliminary analyses for the grant submission, it would have been inappropriate to subsequently preregister the hypotheses. Follow-up research should be pre-registered, as is best practice. We were unable to test a full mediation model testing whether weight gain predicts worse same-day behavioral outcomes via negative postweighing cognitions. Unfortunately, this study’s sample size would not adequately power the multilevel mediation necessary for this analysis (Pan et al., 2018). Future work should explore this question within a larger sample with sufficient power. The small sample may also have been underpowered to detect small cross-level interaction effects, and more significant findings may have emerged for the tests of Hypothesis 1b had the sample been larger. Future studies should examine these relationships over a longer period of time, given the short (5-day) study timeframe. Many participants completed the morning and nightly surveys too late following the prompt, which led to exclusion from analyses, and may have biased results. Future research should consider investing in integrating smart scales with EMA platforms, such that EMA surveys can triggered in response to weigh-ins. Future work should also examine how long negative responses to weighing last, perhaps using more frequent assessments throughout the day. It is unclear whether maladaptive cognitions lasted all day, or whether they simply set into motion a day of overeating and poor exercise. The persistence of these negative cognitions has important implications for when interventions can and should be administered.

The sample size was small, and results may not generalize to outside the US (e.g., to other ethnicities) or to men. The study was a secondary analysis of a parent clinical trial and was therefore constrained by that sample. Women are highly overrepresented in BWL programs (Pagoto et al., 2012; Robertson et al., 2017), including the parent clinical trial. Of note, men and women have equal outcomes in these programs (Sauder et al., 2021). Women tend to have higher internalized weight bias (Boswell & White, 2015) and experience shame (Else-Quest et al., 2012) more than men, and therefore may struggle with weight-related setbacks more than men. Research on men’s responses to weight gain and weight-related setbacks, as well as their overall experience with behavioral weight loss attempts, is warranted.

Participants had varying weight loss goals, and many in the study hoped to maintain their weight, whereas others hoped to lose more. Although we attempted to account for this variability by examining weight loss satisfaction far, we could not explore whether these differences depended on objective proximity to the participants’ ultimate weight goal. There were also limitations of the measurements. The validity of self-report EMA items is understudied and warrants more research (Stinson et al., 2022). Although similar measures of negative affect and shame have been used in past studies (Asselbergs et al., 2016; Nechita & David, 2023), the measures of confidence in the effectiveness of weight control and motivation were created for the current study. Additionally, the measurement of calorie intake used in this study asked individuals self-report their daily intake relative to their calorie goal, which is imprecise and susceptible to self-report bias (although, this is a limitation of all self-reported measurements of calorie intake, which are frequently used in eating behavior research (Carpenter et al., 2022). Finally, we dichotomized weight gain into weight gain vs. loss/maintenance, which could not distinguish the size of the weight gain or loss. However, we also ran these analyses using a continuous measure of weight change, and patterns were largely the same. Concerns about careless responding on surveys are minimized due to the extensive enrollment procedures in the parent trial that resulted in a highly conscientious sample.

## Conclusions

This study is the first to provide real-time quantitative data on the post-weighing experience of those engaged in BWL and to show that these post-weighing responses are clinically relevant, influencing weight control behavior that day. Results show that guilt/shame and low motivation can occur in response to weight gain and lead to less effective weight control behavior that day. Although the current study only examined daily changes in weight gain, the relationships found here may be evidence of the start of a vicious cycle of demoralization and weight regain. Using JITAIs to promote more adaptive responses in the moments after a weigh-in may disrupt this negative cycle.

## Figures and Tables

**Table 1:** Correlation table: between and within-person associations

	1	2	3	4	5	6	7	8	9	10	11	Between-Person Mean	SD	Range
**1. Overall Weight Change (%)^a^**	*	−0.75**	−0.08	−0.48**	0.64**	−0.34*	−0.36*	−0.14	0.21	−0.48**	−0.33**	−0.10	0.07	
**2. Overall Weight Satisfaction**		*	−0.02	0.43**	−0.75**	0.34*	0.62**	0.29*	−0.23	0.51**	0.37*	6.42	2.28	1–10
**3. Gained Weight (0 = no, 1 = yes)**			*	0.02	−0.08	−0.02	0.00	−0.29*	0.33*	0.43**	0.11	1.60	1.12	0–5
**4. Mood**			−0.23**	*	−0.77**	0.48**	0.52**	0.31*	−0.24	0.40**	0.14	7.16	1.43	1–10
**5. Guilt/Shame**			0.20**	−0.75**	*	−0.38**	−0.69**	−0.41**	0.30*	−0.53**	−0.36**	4.66	1.78	1–10
**6. Confidence in Weight Control**			−0.07	0.43**	−0.40**	*	0.47**	0.12	−0.10	0.27	0.12	4.07	0.74	1–5
**7. Motivation for Weight Control**			−0.03	0.48**	−0.63**	0.39**	*	0.49**	−0.25	0.54**	0.14	3.32	0.94	1–5
**8. Perceived Weight Control Behavior**			0.21*	−0.22**	0.32**	0.11	−0.22*	*	−0.63**	0.31*	0.25	2.08	0.48	1–5
**9. Perceived Calorie Intake**			−0.07	−0.16	0.28**	−0.02	−0.18	−0.58**	*	−0.06	−0.01	3.48	0.91	1–5
**10. Calorie Tracking (0 = no, 1 = yes)**			0.03	0.27**	−0.42**	0.30**	0.45**	0.30**	0.17*	*	0.32*	2.34	1.91	0–5
**11. Moderate-to-Vigorous Physical Activity**			−0.03	0.13	−0.24**	0.05	0.13	0.26**	0.04	0.17*	*	34.91	32.85	
**Intraclass Correlation Coefficient**				0.51	0.78	0.74	0.59	0.25	0.36		0.44			

In between person correlations, dichotomous variables are the sum of all five days

*Note* Correlations for between-person associations are in the upper triangle. Within-person correlations are in the lower triangle, shaded in grey

** Indicates p < .01,

* Indicates p < .05

**Table 2:** Themes from open-ended responses to the question “How do you feel about your weight right now?” immediately after participants weighed themselves

Theme	Overall frequency	Frequency on days with weight gain	Frequency on days with weight loss or maintenance	Exemplar quotes
**Room for Improvement**	15/177 (8.5%)	10/80 (12.5%)	5/97 (5.2%)	*“Slow and steady. It’s never fast enough, but at least it’s going down.”*
**Neutral**	43/177 (24.3%)	28/80 (35.0%)	15/97 (15.5%)	*“I feel neutral I’m not happy but I’m not upset.”* *“It’s what I anticipated and I feel fine.”*
**Pleased/Good Mood**	92/177 (52.0%)	19/80 (23.8%)	73/97 (75.3%)	*“I feel great, I had lost weight.”* *“Positive. My weight was down a few more ounces today, which motivates me to stay on track.”*
**Hopeful**	6/177 (3.4%)	3/80 (3.8%)	3/97 (3.1%)	*“I am confident that I will be able to maintain my weight loss and be able to recover from temporary setbacks.”*
**Surprised**	8/177 (4.5%)	1/80 (1.3%)	7/97 (7.2%)	*“I was expecting it to be a lot more, so I feel relieved that I am not as far off track as I thought I was!”*
**Negative Mood**	36/177 (20.3%)	29/80 (36.3%)	7/97 (7.2%)	*“I feel terrible, it showed 3 pounds gain in one day.”* *“Not good. I have gained weight and I’m upset with myself.”* *“I’m not happy. Up 2.5 lbs in 2 days. I did NOT consume 8750 more calories than I burned. This always seems to happen when I travel and I do not understand it!”*

## Data Availability

Data and code for all analyses are stored and available to the public at Open Science Framework (https://osf.io/bk7m8/?view_only=0eba75a085da4442b00746d3ab599c4d).

## Footnotes

1 To examine multivariate outliers, we used the “influence” statement in SAS PROC MIXED to identify Cook’s Distances (Schabenberger, 2005). We used 0.08 as the Cook’s Distance cutoff (i.e., 4/n, n = the grouping factor, in this case, 50 participants (Nieuwenhuis et al., 2012). We identified up to 5 potential outliers for some analyses. However, we reran the results while excluding these outliers, and there were no changes to the patterns of results reported here.

2 When the three MVPA outliers (> 3 SD above the mean) were included in the analysis, mood was not predictive of MVPA (*b* = 2.15, *SE* = 1.92, *t* = 1.12, *p* = .27).

3 When the three MVPA outliers (> 3 SD above the mean) were included in the analysis, guilt/shame was only marginally predictive of MVPA (*b*=−3.60, *SE* = 2.02, *t*=−1.78, *p* = .08).
